# Preoperative chemoradiation-induced hematological toxicity and related vertebral dosimetry evaluations in patients with locally advanced gastric cancer: data from a phase III clinical trial

**DOI:** 10.1186/s13014-023-02269-6

**Published:** 2023-06-09

**Authors:** Ji-jin Wang, Han Shao, Li Zhang, Ming Jing, Wen-jing Xu, Heng-wen Sun, Zhi-wei Zhou, Yu-jing Zhang

**Affiliations:** 1 Department of Radiation Oncology, State Key Laboratory of Oncology in South China, Collaborative Innovation Center for Cancer Medicine, Sun Yat-sen University Cancer Center, 510060 Guangzhou, People’s Republic of China; 2 Department of Oncology, Renmin Hospital, Hubei University of Medicine, 442000 Shiyan, People’s Republic of China; 3 Department of Radiotherapy, Guangdong Provincial People’s hospital, Guangdong Academy of Medical Sciences, Cancer center, 510080 Guangzhou, People’s Republic of China; 4 Department of Gastric Surgery, State Key Laboratory of Oncology in South China, Collaborative Innovation Center for Cancer Medicine, Sun Yat-sen University Cancer Center, 510060 Guangzhou, People’s Republic of China

**Keywords:** Vertebral body, Dosimetric parameter, Hematologic toxicity, Gastric, Chemoradiotherapy

## Abstract

**Background:**

To explore the hematological toxicity (HT) induced by neoadjuvant chemoradiotherapy (nCRT) compared with neoadjuvant chemotherapy (nCT) and to identify the appropriate vertebral body (VB) dosimetric parameters for predicting HT in patients with locally advanced gastric cancer (GC).

**Methods:**

In the phase III study, 302 patients with GC from an ongoing multi-center randomized clinical trial (NCT 01815853) were included. Patients from two major centers were grouped into training and external validation cohorts. The nCT group received three cycles of XELOX chemotherapy, while the nCRT received the same dose-reduced chemotherapy plus 45 Gy radiotherapy. The complete blood counts at baseline, during neoadjuvant treatment, and in the preoperative period were compared between the nCT and nCRT groups. The VB was retrospectively contoured and the dose-volume parameters were extracted in the nCRT group. Patients’ clinical characteristics, VB dosimetric parameters, and HTs were statistically analyzed. Instances of HT were graded according to the Common Terminology Criteria for Adverse Events v5.0 (CTCAE v5.0). The receiver operating characteristic (ROC) curves were generated to identify the optimal cut-off points for dosimetric variables and verify the prediction efficiency of the dosimetric index in both training and external validation cohorts.

**Results:**

In the training cohort, 27.4% Grade 3 + HTs were noted in the nCRT group and 16.2% in the nCT group (*P* = 0.042). A similar result was exhibited in the validation cohort, with 35.0% Grade 3 + HTs in the nCRT group and 13.2% in the nCT group (*P* = 0.025). The multivariate analysis of the training cohort revealed that V_5_ was associated with Grade 3 + leukopenia (*P* = 0.000), Grade 3 + thrombocytopenia (*P* = 0.001), and Grade 3 + total HTs (*P* = 0.042). The Spearman correlation analysis identified a significant correlation of V_5_ with the white blood cell nadir (*P* = 0.0001) and platelet nadir (*P* = 0.0002). The ROC curve identified the optimal cut-off points for V_5_ and showed that V_5_ < 88.75% could indicate a decreased risk of Grade 3 + leukopenia, thrombocytopenia, and total HTs in the training as well as the external validation cohorts.

**Conclusions:**

Compared with nCT, nCRT could increase the risk of Grade 3 + HT in patients with locally advanced GC. Dose constraints of V_5_ < 88.75% in irradiated VB could reduce the incidence of Grade 3 + HT.

## Background

Gastric cancer (GC) is the third leading cause of cancer-related deaths worldwide, with > 40% of new cases being detected in China [1]. Surgical resection with perioperative chemotherapy is a standard treatment for most cases of locally advanced GC [2, 3]. In the absence of positive results from adjuvant chemoradiotherapy studies [4, 5], there is increasing expectation of neoadjuvant chemoradiotherapy (nCRT) for improving survival. Several prospective randomized clinical trials have compared nCRT with neoadjuvant chemotherapy (nCT) for GC and esophagogastric junction (EGJ) adenocarcinoma, including the published POET [6] and Neo-AEGIS trials [7], and the ongoing TOPGEAR [8], CRITICS-II [9], PREACT [10], and the Neo-CRAG trials [11]. Most published results have not shown a particularly increase in hematological toxicity (HT) in the nCRT arm than in the nCT arm; for example, the TOPGEAR interim results showed that 52% of Grade 3 + HTs occurred in the nCRT group and 50% occurred in the nCT group [8]. However, tumor location and pattern of lymphatic extension can influence the characteristics of the radiation field and theoretically, the addition of a major treatment modality can potentially increase the risk of adverse effects.

Previous studies have reported the relationship between HT and bone marrow radiotherapy dosimetry, though the majority of them were about pelvic bone irradiation in cervical cancer and colorectal cancer [12, 13]. Similar studies have also been performed on radiotherapy for esophageal and pancreatic cancers [14, 15]. In radiotherapy for GC and EGJ cancer, vertebral bone marrow can be considerably affected given its anatomical adjacency to the clinical target volume (CTV) and the necessity for sparing the lateral organs at risk (OARs), such as the kidneys, liver, lungs, and heart. Studies have shown that up to 50% of the spinal bone marrow is hematopoietically active [16]. Since bone marrow stem cells are highly radiosensitive, the hematopoietic function of vertebral bone marrow can directly be impaired by radiotherapy for GC [17, 18]. Therefore, it is necessary to explore the influence of dosimetric factors on HT due to nCRT for GC.

The Neo-CRAG trial (NCT 01815853) [11] is a large multi-center phase III clinical trial that has just completed patient recruitment. The inclusion criteria was the most advanced non-metastatic GC (e.g., T3N2-3, T4aN+, T4bNany, and M0), with the lower border of station 16a2 lymph nodes always being included in the CTV of radiotherapy.

In the preliminary data analysis, we noted a trend of increased HT in the nCRT group than in the nCT group, as well as an association between vertebral body (VB) dosimetry and HT grade. Thus, identifying the dosimetric parameters associated with HT could be critical for bone marrow-sparing in radiotherapy planning. Therefore, this study aimed to explore the difference in the risk of HT between nCRT and nCT groups and to identify the best predictive dosimetric factors of HT for locally advanced GC.

## Methods

### Patients

This study was based on an ongoing multi-center randomized controlled phase III clinical study (Neo-CRAG trial, NCT 01815853) that compared nCRT with nCT for patients with locally advanced gastric adenocarcinoma. The inclusion criteria were patients with histologically confirmed gastric adenocarcinoma, at cT3N2-3M0, cT4aN + M0, or cT4bNanyM0 clinical stage, aged 18–75 years, with adequate organ function, and with an Eastern Cooperative Oncology Group performance status score ≤ 2. The clinical stage was based on gastroscopy or endoscopic ultrasound (EUS), computed tomography (CT), and exploratory laparoscopy findings. The primary endpoint was disease-free survival (DFS) and the secondary endpoint was overall survival (OS), pathological complete remission (pCR) rate, and treatment safety. The clinical trial has completed the recruitment of 620 patients as of July 2022.

Among the patients enrolled in the Neo-CRAG trial, we included 302 patients from two major participating centers who completed preoperative therapy between June 2013 and November 2021. The clinical characteristics of these patients are shown in Table 1. Of them, 113 treated with nCRT and 111 treated with nCT at Sun Yat-sen University Cancer Center were grouped under the training cohort, while 40 treated with nCRT and 38 treated with nCT at the Guangdong Provincial People’s Hospital were grouped under the external validation cohort.

**Table 1:** Patients’ clinicopathological and hematological characteristics

Characteristics	Training cohort			Validation cohort		
	nCRT (n = 113)	nCT (n = 111)	*P*	nCRT (n = 40)	nCT (n = 38)	*P*
Sex			0.612			0.157
Female Male	34(30.1%) 79(69.9%)	30(27.0%) 81(73.0%)		8(20.0%) 32(80.0%)	13(34.2%) 25(65.8%)	
Age (years)			0.143			0.341
< 60 ≥ 60	48(42.5%) 65(57.5%)	58(52.3%) 53(47.7%)		19(47.5%) 21(52.5%)	14(36.8%) 24(63.2%)	
Lauren type			0.826			0.567
Intestinal Diffuse Mixed Unknown	48(42.5%) 31(27.4%) 23(20.4%) 11(9.7%)	41(36.9%) 31(27.9%) 27(24.3%) 12(10.8%)		7(17.5%) 11(27.5%) 4(10.0%) 18(45.0%)	9(23.7%) 9(23.7%) 7(18.4%) 13(34.2%)	
Primary tumor site			0.146			0.116
Upper third Middle third Lower third	61(54.0%) 22(19.5%) 30(26.5%)	53(47.7%) 33(29.7%) 25(22.5%)		17(42.5%) 8(20.0%) 15(37.5%)	25(65.8%) 4(10.5%) 9(23.7%)	
WHO histological grade			0.158			0.993
Low Moderate/ High Unknown	57(50.4%) 56(49.6%) 0(0%)	55(49.5%) 52(46.8%) 4(3.7%)		21(52.5%) 13(32.5%) 6(15.0%)	20(52.6%) 12(31.6%) 6(15.8%)	
cTNM stage			0.70			0.104
III IV	96(85.0%) 17(15.0%)	97(87.4%) 14(12.6%)		39(97.5%) 1(2.5%)	33(86.8%) 5(13.2%)	
Hematological toxicity						
Grade 3 + Leukopenia Grade 3 + Neutropenia Grade 3 + Anemia Grade 3 + thrombocytopenia Grade 3 + Total HTs	13(11.5%) 12(10.6%) 16(14.2%) 13(11.5%) 31(27.4%)	1(0.9%) 5(4.5%) 16(14.4%) 0(0%) 18(16.2%)	0.001 0.084 0.956 0.000 0.042	6(15.0%) 6(15.0%) 7(17.5%) 4(10.0%) 14(35.0%)	0(0%) 1(2.6%) 4(10.5%) 0(0%) 5(13.2%)	0.026 0.056 0.376 0.045 0.025
Blood cell nadir value						
WBC (10^9/L) ANC (10^9/L) HGB (g/L) PLT (10^9/L)	3.05**±**0.91 1.82**±**0.68 98.69±16.94 97.08±43.22	4.83±1.67 2.60±1.43 105.96±22.83 156.94±51.88	0.000 0.000 0.008 0.000	3.27±1.23 1.98±0.88 101.98±20.59 122.1±65.47	4.65±1.22 2.28±0.79 114.68±20.05 158.66±37.70	0.000 0.114 0.007 0.003
Pre-WBC (10^9/L)	6.20(5.15–7.37)	6.34(5.14–7.80)	0.430	6.67(5.49–8.14)	6.03(4.80–7.74)	0.210
Pre-ANC (10^9/L)	4.00(3.00-4.90)	3.81(3.00-5.20)	0.961	3.71(2.91–4.68)	3.57(2.59–4.60)	0.484
Pre-HGB (g/L)	122(100–136)	125(104–139)	0.335	130(108–143)	130(119–139)	0.865
Pre-PLT (10^9/L)	286(219–337)	292(230–372)	0.205	283(243–338)	268(236–321)	0.242

nCRT, neoadjuvant chemoradiotherapy; nCT, neoadjuvant chemotherapy; cTNM stage, clinical tumor-node-metastasis stage; HT, hematological toxicity; WBC, white blood cell; ANC, absolute neutrophil count; HGB, hemoglobin; PLT, platelet

### Treatment

As per the trial protocol [11], patients in the nCT group received three 21-day cycles of XELOX chemotherapy (capecitabine 1000 mg/m², bid, d1-14 + oxaliplatin 130 mg/m², d1). Patients in the nCRT group received one cycle of XELOX induction chemotherapy, followed by radiotherapy of 45 Gy in 25 daily fractions, concurrently with two 21-day cycles of dose-reduced XELOX chemotherapy (capecitabine 825 mg/m², bid, d1-14 + oxaliplatin 100 mg/m², d1). Subsequently, radical gastrectomy was performed 3–4 weeks and 6–8 weeks after the conclusion of nCT and nCRT, respectively. The patients then continued to receive three 21-day cycles of adjuvant XELOX chemotherapy (standard dose intensity as in nCT group) 3–4 weeks after surgery.

### Radiation therapy and target volume delineation

Patients in the nCRT group were treated as follows: three-dimensional conformal radiation therapy (3D-CRT) for seven patients (one in the training cohort, six in the validation cohort) and intensity-modulated radiation therapy (IMRT) for the other patients. During the scan, the patients were immobilized in the supine position with a vacuum cushion and scanned with simulation computed tomography (CT-sim), including plain and venous-enhanced scans from T4 to L5 vertebral levels. The inter-slice thickness of the CT scan was 5 mm. About 50–100 mL of prepared iopamidol solution was orally administered as the contrast agent 20 min before and at the time of CT-sim. Patients were required to fast for at least 3 h before the CT-sim and before each radiation treatment to account for inter-fractional variability in gastric distention due to gastric filling. Before the scan, gastroscopy was performed to place titanium clips at the cephalic and caudal edges of the tumor as a fiducial marker for target volume delineation.

The principle of target volume delineation was designed at beginning of the Neo-CRAG trial and was based on preoperative radiation recommendations of the European Organization for Research and Treatment of Cancer (EORTC-ROC) [19, 20], the National Comprehensive Cancer Network (NCCN) guidelines for GC [21], and preoperative chemoradiation studies published the M.D. Anderson Cancer Center [22].

To elaborate, the gross tumor volume of the primary tumor (GTVt) and involved lymph nodes (GTVnd) were delineated based on baseline CT scan, gastroscopy or EUS, and exploratory laparoscopic findings. The CTV included GTVt with a 3 cm mucosal expansion and a 1.0–1.5 cm extragastric expansion, GTVnd with a 5 mm expansion, and elective regional lymphatic drainage regions. The mucosal expansion included the distal esophagus or proximal duodenum when appropriate. The inclusion of adjacent high-risk structures, including the inner half of the left diaphragm, parts of the pancreas, and neighboring parietal peritoneum were also considered. The CTV delineation had to avoid the vertebral body and include no more than 5 mm of liver tissues. The CTV was expanded by 5 mm in three dimensions to decide the planning target volume (PTV).

The location of the primary tumor determined which elective lymph node groups would be included as per the JGCA classification, considering a 5 mm margin around the corresponding vessels:

- Proximal 1/3 stomach and distal EGJ primaries:
  - Essential: groups 1, 2, 3, 4sa, 7, 9, 10 (for neighboring greater curvature tumor), 11p, 12 (for lesser curvature tumor), 16a, 19, 20, 110, and 111.
  - Optional: groups 4sb (for greater curvature tumor), 5, 8, 11d, and 13.
- Middle 1/3 stomach primaries:
  - Essential: groups 1, 2, 3, 4sa, 4sb, 4d, 5, 6, 7, 8, 9, 10, 11p, 11d, 12, 13, and 16a.
  - Optional: groups 14, 17, 18, and 19.
- Distal 1/3 stomach primaries:
  - Essential: groups 1, 3, 4d, 5, 6, 7, 8, 9, 11p, 12, 13, 16a, 17, and 18.
  - Optional: groups 4sb, 10, and 11d.

For dose limitations of the OAR, the liver volume percentage that received ≥ 30 Gy (V_30_) had to be less than 30% (V_30_ < 30%) and the mean dose (D_mean_) had to be < 22–25 Gy. For the duodenum and small intestine, D_mean_ was ≤ 50 Gy and V_45_ < 33%. For the kidneys, V_18_ was < 33% and D_mean_ was ≤ 17 Gy. For the heart, V_40_ was < 30% and V_25_ < 50%. For the spinal cord, the maximum dose (D_max_) was ≤ 40 Gy.

Forward or inverse treatment planning was used for 3D-CRT or IMRT, respectively, based on modern Monaco (Elekta, Crawley, UK), Eclipse (Varian Medical Systems, Palo Alto, CA, USA), or Pinnacle (Philips Medical Systems, Madison, WI, USA) treatment planning systems, using the direct machine parameter optimization algorithm.

### Vertebral body target delineation

**Fig. 1:**
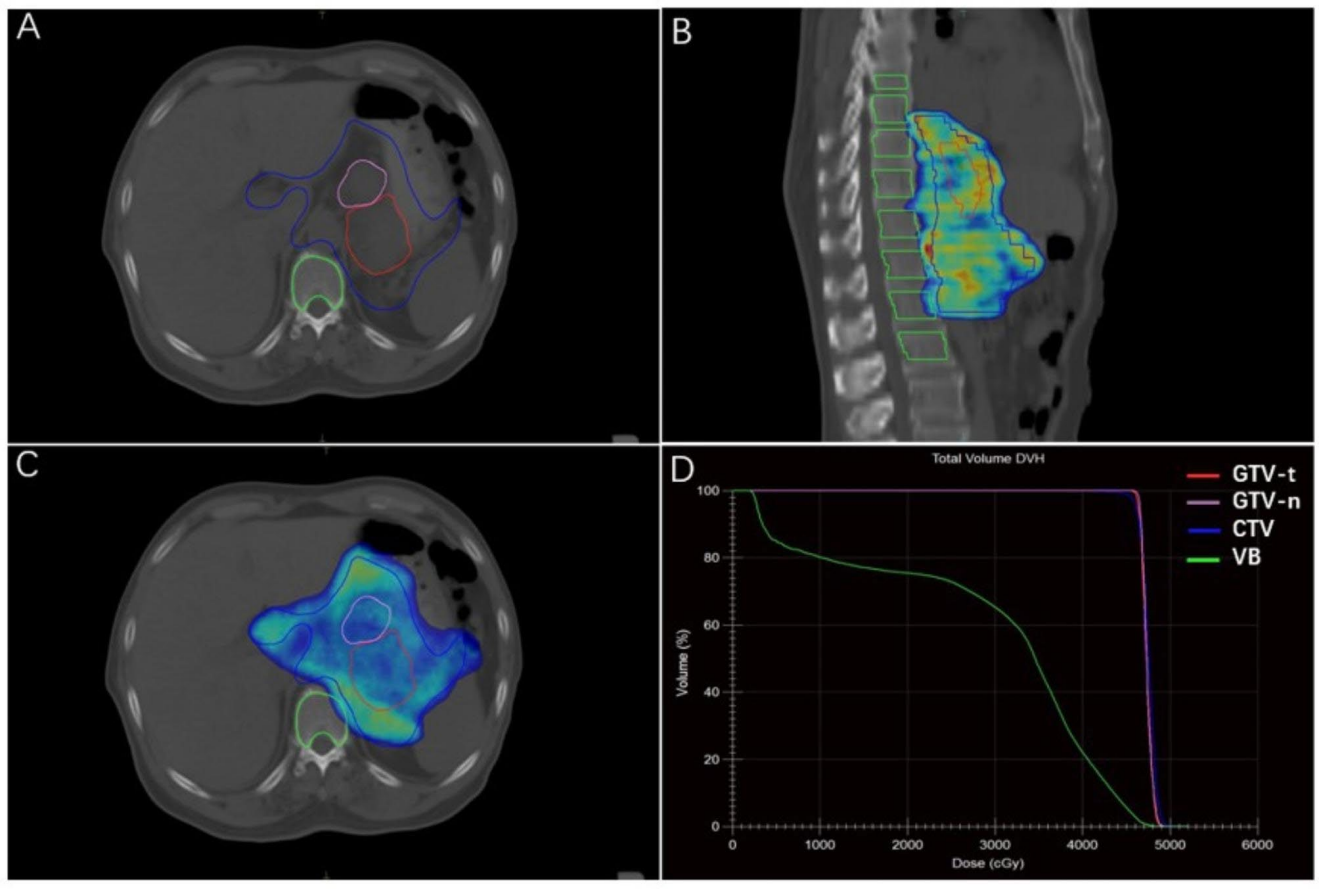
Delineation of the vertebral body and dose-volume histogram for a radiation treatment plan. (**A**) Transverse section; (**B**) Sagittal section; (**C**) Transverse section with dose coverage; (**D**) The Dose-Volume Histogram (DVH)

As illustrated in Fig. 1, the VB was retrospectively delineated manually based on CT simulation. The unified protocol of VB delineation was designed as follows: (a) VB and anterior pedicle were delineated, not including the vertebral appendages. The boundary of pedicle delineation was at the horizontal line across the center of the vertebral foramen. (b) The window width/level was set at W1600 Hu/L400 Hu to share the advantages of both the bone and mediastinal windows. (c) Only the cancellous bone was delineated and the cortical bone was avoided. (d) The upper boundary of the delineated VB was 2 cm above the CTV and the lower boundary was 3 cm below the CTV, not counting the intervertebral discs (IVD). The thoracic IVD thickness was set as 0.5 cm (one slice) and the lumbar IVD thickness as 1 cm (two slices). All the VB contours were delineated under the guidance of one single chief physician.

After delineation, a dose-volume histogram (DVH) was obtained from the original treatment plan. The dosimetric parameters of VB including the total vertebral body volume (VBV), D_max_, D_mean_, minimum dose (D_min_), and the volume percentage of dose receiving ≥ x Gy (V_5_, V_10_, V_20_, V_30_, V_40_, and V_45_) were recorded.

### Hematological toxicity evaluation

Routine blood investigations were regularly performed for all patients. Complete blood counts including white blood cell (WBC), neutrophil (NEU), hemoglobin (HGB) and platelet (PLT) were determined at the time of diagnosis and weekly during neoadjuvant therapy and pre-operative evaluation. Instances of HT were graded according to the Common Terminology Criteria for Adverse Events v5.0 (CTCAE v5.0).

### Statistical analysis

The clinicopathological characteristics, hematological indicators, and dosimetric parameters were evaluated by t-test or Chi-squared test or Fisher’s exact test, when appropriate. Univariate (UVA) and multivariate (MVA) logistic regression was performed to identify dosimetric variables associated with the development of Grade3 + HT. Spearman correlation analysis was used to test correlations between dosimetric variables and absolute blood cell nadirs. Receiver Operating Characteristic (ROC) curves with the Youden method were used to identify the optimal dose-volume thresholds of predictive dosimetric parameters and to evaluate the predictive efficiency of the cutoff value both in the training cohort and the external validation cohort.

All statistical analyses were performed with SPSS software 23.0, and *P* < 0.05 was considered to be significant. GraphPad Prism 8.0 and RStudio version 4.1.2 were used for plotting.

## Results

### Comparison of clinicopathological characteristics between the nCRT and nCT groups

The clinicopathological characteristics including sex (*P =* 0.612), age (*P =* 0.143), Lauren type (*P =* 0.826), primary tumor site (*P =* 0.146), histological grade (*P =* 0.158), and cTNM stage (*P =* 0.70) were not significantly different between the nCRT and nCT groups in the training cohort (Table 1). Similarly, there were no significant differences between the two groups in the validation cohort.

### Differences in HT between the nCRT and nCT groups

There was no difference of pretreatment WBC (pre-WBC), pre-NEU, pre- HGB, and pre-PLT between nCRT group and nChT group both in the training and validation cohort (Table 1).

**Fig. 2:**
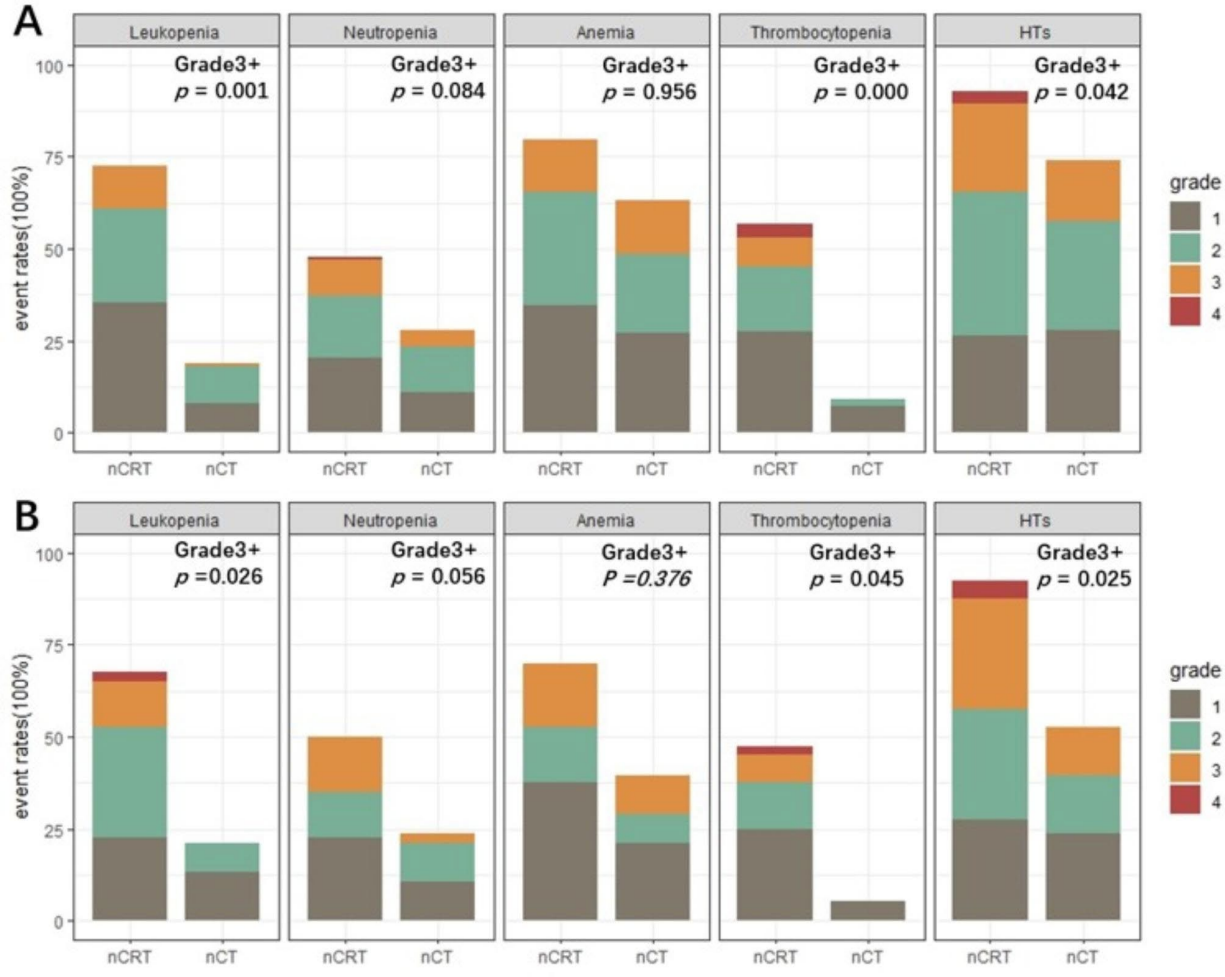
Different grades of hematological toxicity in the training and validation cohorts. (**A**) Training cohort: Grade 3 + Leukopenia (*P* = 0.001), thrombocytopenia (*P* = 0.000), and total HTs (*P* = 0.042) showed great significance between nCRT and nCT group; (**B**) External validation cohort: Grade 3 + Leukopenia (*P* = 0.026), thrombocytopenia (*P* = 0.045), and total HTs (*P* = 0.025) showed great significance between nCRT and nCT group

Patients in the nCRT group experienced more Grade 3 + leukopenia (11.5% vs. 0.9%, *P =* 0.001), Grade 3 + thrombocytopenia (11.5% vs. 0%, *P =* 0.000), and Grade 3 + total HTs (27.4% vs. 16.2%, *P =* 0.042) compared with patients in the nCT group (Table 1). The incidences of Grade 3 + neutropenia (*P =* 0.084) and Grade 3 + anemia (*P =* 0.956) were not significantly different between the two groups. Grade 4 HT was rare and Grade 5 HT did not occur during the entire neoadjuvant treatment period. In the validation cohort, patients who received nCRT also suffered more Grade 3 + HTs (*P =* 0.025) than those in the nCT group. The different grades of HT in the training and external validation cohorts are shown in detail in Fig. 2.

Further, the influence of the addition of radiotherapy on blood cell nadirs was explored (Table 1). The mean ± standard deviation (SD) nadir counts of white blood cell (WBC) count, absolute neutrophil count (ANC), hemoglobin (HGB), and platelet (PLT) in the nCRT group were 3.05 ± 0.91*10^9^/L, 1.82 ± 0.68*10^9^/L, 98.69 ± 16.94 g/L, and 97.08 ± 43.22*10^9^/L, respectively. The corresponding blood cell nadirs in the nCT group were 4.83 ± 1.67*10^9^/L, 2.60 ± 1.43*10^9^/L, 105.96 ± 22.83 g/L, and 156.94 ± 51.88*10^9^/L, respectively. Patients in the nCRT group had significantly lower WBC nadir (*P =* 0.000), ANC nadir (*P* = 0.000), HGB nadir (*P* = 0.008), and PLT nadir (*P* = 0.000) compared with those in the nCT group. In the validation cohort, the difference in the blood cell nadirs between the two groups showed the same trend as that in the training cohort.

### Modeling dosimetric predictors of hematological toxicity in the training cohort

The VB radiation dosimetric parameters of all patients treated with nCRT from two centers are summarized in Table 2. Only V_30_ (*P* = 0.049) was significantly different between the training and validation cohorts, while the other dose-volume parameters were not.

**Table 2:** The vertebral body dosimetric parameters (mean ± SD)

Parameters	Training cohort (n = 113)	Validation cohort (n = 40)	*P*
VBV (cm³) Dmin (cGy) Dmax (cGy) Dmean (cGy) V_5_ (%) V_10_ (%) V_20_ (%) V_30_ (%) V_40_ (%) V_45_ (%)	131 ± 27.8 179.7 ± 78.7 4834.7 ± 158.6 2830.5 ± 313.3 85.3 ± 5.4 79.9 ± 5.7 71.9 ± 6.9 58.3 ± 10.7 26.4 ± 11.3 9.1 ± 6.5	120.6 ± 31.3 240.8 ± 81.3 4740.7 ± 140.7 2624.4 ± 338.4 87.3 ± 7.2 78.9 ± 5.4 67.8 ± 8.9 49.4 ± 13.0 17.4 ± 12.0 4.3 ± 5.7	0.264 0.368 0.307 0.358 0.832 0.943 0.367 0.049 0.562 0.121

SD, standard deviation; VBV, vertebral body volume; D_min_, minimum dose; D_max_, maximum dose; D_mean_, mean dose; V_x_, the volume percentage of dose receiving ≥ x Gy

**Fig. 3:**
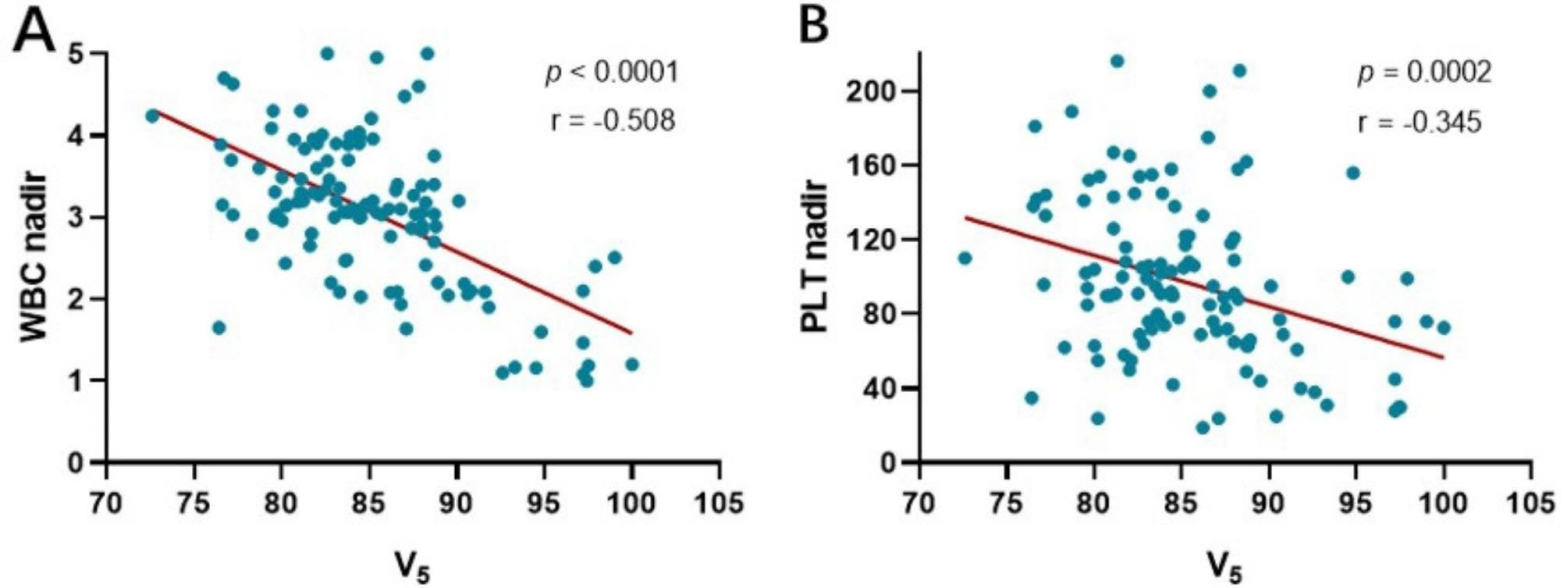
Spearman correlation analysis between V_5_ and blood cell nadirs in the training cohort. (**A**) Increased V_5_ was correlated with lower WBC nadir (r = -0.508, *P* < 0.0001); (**B**) Increased V_5_ was correlated with lower PLT nadir (r = -0.345, *P* = 0.0002)

The dose-volume metrics of the VB were entered into UVA in the training cohort. The results showed that increased D_min_, D_mean_, V_5_, V_10_, V_20_, V_30_, and V_40_ were risk factors for Grade 3 + leukopenia; increased V_5_ and V_10_ were risk factors for Grade 3 + thrombocytopenia; and D_max_ and V_5_ were risk factors for Grade 3 + total HTs. Subsequently, variables with *P* ≤ 0.15 in the UVA were included in the MVA. The results showed that V_5_ was a risk factor for Grade 3 + leukopenia (odds ratio [OR]: 1.353, 95% confidence interval [CI]: 1.179–1.553, *P* = 0.000) and Grade 3 + thrombocytopenia (OR: 1.192, 95% CI: 1.074–1.324, *P =* 0.001). Meanwhile, V_5_ also seems like a risk factor for Grade 3 + total HTs (OR: 1.086, 95% CI: 0.987–1.195, *P =* 0.042), as the statistics (OR and 95% CI) are at a critical value. Table 3 detailed the results of UVA and MVA. Spearman correlation analysis revealed that increased V_5_ was correlated with a lower WBC nadir (r = -0.508, *P* < 0.0001) and lower PLT nadir (r = -0.345, *P* = 0.0002) (Fig. 3).

**Table 3:** Univariate and multivariate logistic regression analysis

	**UVA**		MVA	
	OR (95% CI)	*P*	OR (95% CI)	*P*
**Grade 3+ Leukopenia**				
V_5_ (%) V_10_ (%) V_20_ (%) V_30_ (%) V_40_ (%) V_45_ (%)	1.353 (1.179–1.553) 1.287 (1.133–1.462) 1.171 (1.057–1.297) 1.071 (1.002–1.145) 1.041 (0.986–1.098) 1.037 (0.951–1.132)	0.000 0.000 0.003 0.043 0.147 0.411		
V_5_ (%) V_10_ (%) V_20_ (%) V_30_ (%) V_40_ (%) V_45_ (%)	1.353 (1.179–1.553) 1.287 (1.133–1.462) 1.171 (1.057–1.297) 1.071 (1.002–1.145) 1.041 (0.986–1.098) 1.037 (0.951–1.132)	0.000 0.000 0.003 0.043 0.147 0.411	1.353 (1.179–1.553)	0.000
**Grade 3 + Thrombocytopenia**				
VBV (cm³) Dmin (cGy) Dmax (cGy) Dmean (cGy)	1.004 (0.985–1.024) 1.004 (0.998–1.010) 0.999 (0.996–1.003) 1.000 (0.998–1.002)	0.673 0.188 0.607 0.787		
V_5_ (%) V_10_ (%) V_20_ (%) V_30_ (%) V_40_ (%) V_45_ (%)	1.192 (1.074–1.324) 1.115 (1.015–1.244) 0.996 (0.919–1.081) 0.986 (0.937–1.037) 0.986 (0.938–1.036) 0.964 (0.875–1.061)	0.001 0.022 0.930 0.583 0.577 0.452	1.192 (1.074–1.324)	0.001
**Grade 3 + HTs**				
VBV (cm³) Dmin (cGy) Dmax (cGy) Dmean (cGy)	1.003 (0.985–1.023) 1.003 (0.998–1.009) 0.996 (0.993-1.000) 0.999 (0.997–1.001)	0.719 0.274 0.055 0.276		
V_5_ (%) V_10_ (%) V_20_ (%) V_30_ (%) V_40_ (%) V_45_ (%)	1.077 (0.982–1.182) 1.044 (0.955–1.141) 0.985 (0.912–1.063) 0.967 (0.923–1.014) 0.967 (0.921–1.015) 0.968 (0.884–1.059)	0.117 0.346 0.692 0.164 0.173 0.474	1.086 (0.987–1.195)	0.042

UVA, univariate logistic regression; MVA, multivariate logistic regression; OR, odds ratio; CI, confidence interval; HT, hematological toxicity

**Fig. 4:**
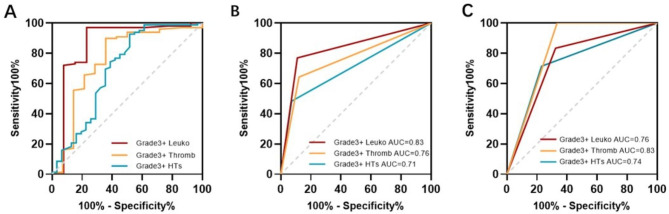
(**A**) ROC curves showing optimal thresholds of vertebral body V_5_ for Grade 3 + leukopenia, Grade 3 + thrombocytopenia, and Grade 3 + total HTs. The optimal cutoff value were V_5_ < 91.7% (AUC = 0.86, *P* < 0.0001), V_5_ < 89.2% (AUC = 0.76, *P* < 0.0001), V_5_ < 88.75% (AUC = 0.70, *P* = 0.0014), respectively; (**B**) The predictive efficiency of V_5_ < 88.75% for Grade 3 + leukopenia (AUC = 0.83), Grade 3 + thrombocytopenia (AUC = 0.76), and Grade 3 + total HTs (AUC = 0.71) in the training cohort; (**C**) The predictive power of V_5_ < 88.75% for Grade 3 + leukopenia (AUC = 0.76), Grade 3 + thrombocytopenia (AUC = 0.83), and Grade 3 + total HTs (AUC = 0.74) in the external validation cohort

Based on the results of MVA, the ROC curve was generated to identify the optimal cut-off values of V_5_ for predicting different HTs. V_5_ < 91.7% (area under the curve [AUC] = 0.86, *P* < 0.0001), V_5_ < 89.2% (AUC = 0.76, *P =* 0.0018), and V_5_ < 88.75% (AUC = 0.70, *P =* 0.0014) were the optimal cut-off values for predicting Grade 3 + leukopenia, Grade 3 + thrombocytopenia, and Grade 3 + total HTs, respectively (Fig. 4A).

### Validation of the predictive efficiency of the best cut-off value

Considering the safety and convenience of clinical application, a lower dose-volume metric of V_5_ < 88.75% was chosen as the best cut-off value for decreasing Grade 3 + HT. As shown in Fig. 4B, V_5_ < 88.75% showed a satisfactory predictive ability for a lower incidence of Grade 3 + leukopenia (AUC = 0.83), Grade 3 + thrombocytopenia (AUC = 0.76), and Grade 3 + total HTs (AUC = 0.71) in the training cohort. Limiting VB V_5_ < 88.75% could reduce the rate of Grade 3 + leukopenia, Grade 3 + thrombocytopenia, and Grade 3 + total HTs by 44.3%, 37.5%, and 54%, respectively.

Furthermore, as is shown in Fig. 4C, we tested the predictive power of V_5_ < 88.75% in the external validation cohort of 40 patients who received nCRT. The AUCs for Grade 3 + leukopenia, Grade 3 + thrombocytopenia, and Grade 3 + total HTs were 0.76, 0.83, and 0.74, respectively, which indicates good predictive ability. The rates of Grade 3 + leukopenia, Grade 3 + thrombocytopenia, and Grade 3 + total HTs decreased by 6.3%, 14.6%, and 14.6%, respectively.

## Discussion

The present study explored the difference in the incidence of HT between nCRT and nCT. Additionally, it investigated the vertebral radiation dosimetric parameters associated with HT in patients with locally advanced GC. To our knowledge, this is the first detailed report characterizing VB dose-volume parameters and their association with HT in GC treated with nCRT. The results demonstrated that the HT of preoperative chemoradiotherapy could be higher than that of preoperative chemotherapy and limiting VB (V_5_ < 88.75%) could decrease the risk of Grade 3 + HT in GC.

The ARTIST trial—the representative study of adjuvant chemoradiotherapy versus adjuvant chemotherapy—showed that Grade 3 + neutropenia occurred in 48.4% of the patients in the postoperative chemoradiotherapy arm and 40.7% of those in the postoperative chemotherapy arm. Further, the rate of Grade 3 + thrombocytopenia was 0.9% in the postoperative chemoradiotherapy arm while none occurred in the postoperative chemotherapy arm [23]. Until now, the TOPGEAR trial is the only large phase III study that has reported a difference in HT in neoadjuvant treatment for GC. The interim results showed that Grade 3 + neutropenia, anemia, thrombocytopenia, and total HTs in the nCRT group reached 45%, 5%, 2%, and 52%, respectively, while the corresponding results in the nCT group were 40%, 7%, 3%, and 50%, respectively [8]. Despite the different rates of HT between these two trials, the frequency of Grade 3 + total HTs was not higher in the nCRT group than in the nCT group. A similar trend of different rates of HT between the two groups was also observed in the ARTIST-2 trial [5]. The CRITICS trial even reported that patients experienced less Grade 3 + neutropenia in the nCRT group (4.4%) than in the nCT group (27%) [24]. Further details of HT differences between chemoradiotherapy and chemotherapy in the above four clinical trials are summarized in Table 4. However, there was no report of HT in the POET trial [6]. Compared with the above studies, this study reported more Grade 3 + leukopenia, thrombocytopenia, and total HTs in the nCRT group than in the nCT group.

**Table 4:** Comparison of hematological toxicity between chemoradiotherapy and chemotherapy reported in major clinically trials of GC

Study (Ref.), Year	Cancer type	Treatment group and HT
ARTIST Trial (4), 2004	GC, Adjuvant therapy, pTNM stage II–III, with node-positive	XP (n = 226) XP-RT (n = 227)
		Grade 3 + neutropenia 40.7% 48.4% Grade 3 + anemia 1.7% 0.4% Grade 3 + thrombocytopenia 0 0.9%
CRITICS Trial (24), 2007	GC and EGJC, Adjuvant therapy, pTNM stage IB–IVA	ChT (n = 233) CRT (n = 245)
		Grade 3 + leukopenia <1.0% 1.0% Grade 3 + neutropenia 27.0% 4.4% Grade 3 + thrombocytopenia <1.0% 1.3%
TOPGEAR Trial interim results (8), 2009	GC, Neoadjuvant therapy, cTNM stage IB–IIIC	ECF/ECX (n = 60) ECF/ECX-RT (n = 60)
		Grade 3 + neutropenia 40.0% 45.0% Grade 3 + anemia 7.0% 5.0% Grade 3 + thrombocytopenia 3.0% 2.0% Grade 3 + total HTs 50.0% 52.0%
ARTIS-2 Trial (5), 2013	GC, Adjuvant therapy, pTNM stage II–III, with node-positive	S-1 (n = 182) SOX (n = 180) SOX-RT (n = 181)
		Grade 3 + neutropenia 1.0% 3.0% 3.0% Grade 3 + anemia 5.0% 8.0% 7.0% Grade 3 + thrombocytopenia 0 3.0% 0

EGJC, esophagogastric junction cancer; XP, capecitabine and cisplatin; RT, radiotherapy; ChT, chemotherapy; CRT, chemoradiotherapy; ECF, epirubicin, cisplatin, and 5-fluorouracil; ECX, epirubicin, cisplatin, and capecitabine; SOX, S-1 and oxaliplatin

The ARTIST and ARTIST-2 studies enrolled GC patients with clinical or pathological stage II and the CRITICS and TOPGEAR studies enrolled patients even with stage IB [8, 24], Meanwhile, we only enrolled patients with more advanced clinical stages of GC (III–IVA). In our study, the lymphatic group 16a_2_, the lower border of which is located at the lower margin of the left renal vein, was included in the CTV of all patients; thus, the delineated target volume was longer and wider. The larger the target volume, the more radiation enters or passes through the unprotected organs, which also aggravates radiation exposure to the VB. More advanced clinical stages and larger target volumes may contribute to the higher HT in the nCRT group in our study.

Though it is well known that radiation to the bone marrow can cause myelosuppression, VB is not yet considered an OAR in major radiotherapy guidelines including those for GC [20, 25–27]. Prior studies reported that VB dosimetric parameters can predict radiation-induced HT in pancreatic and lung cancers [15, 28]. In radiotherapy for GC, to protect the important OARs including the kidneys and liver, the radiation beam has to be arranged along the opposed anteroposterior direction, resulting in high irradiation of the VB. The stomach and its lymphatic drainage region are adjacent to VB, therefore, delineation of the target volume may have a greater impact on the VB in GC than that in the lung or pancreatic cancers. Totally, it is important to protect the VB and set the best VB dose parameters during radiotherapy for GC.

The VB dosimetric studies by Fabian et al. [29], Lee et al. [30], and Zhang et al. [14] focused on esophageal cancer treated with nCRT. The radiotherapy doses were 50.4–59.4 Gy, 41.4–48 Gy, and 41.4–70 Gy, respectively. The first study reported that V_30_ < 14% could reduce the risk of Grade 3 + HT. The second concluded that V_10_ < 77%, V_20_ < 70%, and D_mean_ < 25.9 Gy could decrease the occurrence of Grade 3 + leukopenia. Zhang’s study also demonstrated the correlation of Grade 3 + leukopenia with V_10_, V_20_, and D_mean_. In the above three studies, the differences in their predictive index may be due to differences in their prescribed dose and target volume delineation. Wang et al. [31] found that 20% of patients experienced Grade 3 + HT during capecitabine-based CRT in locally advanced GC and recommended dose constraints of VB V_5_ ≤ 90% to avoid Grade 3 + leukopenia, V_20_ ≤ 78% to avoid Grade 3 + neutropenia, and V_30_ ≤ 60% to avoid Grade 3 + thrombocytopenia during adjuvant CRT. However, the limited sample size of 25 patients in their study makes it challenging to draw conclusive findings. Thus, it is recommended that the dosimetric predictors of Grade 3 + HT be re-evaluated in a larger patient cohort. The vertebral body dosimetric parameters for some common thoracic and abdominal tumors were displayed in Table 5. In the present study, 113 patients with GC who received nCRT in the training cohort were recruited, and the results showed that higher Dmin, Dmean, V_5_, V_10_, V_20_, and V_30_ significantly increased HT of different types. V_5_ was the only independent dosimetric factor predictive of Grade 3 + leukopenia and Grade 3 + thrombocytopenia in subsequent multivariate analyses, and also seemed like a predictor of Grade 3 + total HTs. However, Cheng et al. [32] reported that mean dose and low-dose radiation parameters (V_5_, V_10_, V_15_, V_20_) of whole bone or bone cavities of lumbosacral spine were correlated most significantly with Grade 3 + HT in squamous cell carcinoma of the anal canal. Kumar et al. [33] concluded that Grade 4 HT was associated with lower pelvis V_5_ > 95% in cervical cancer. Meanwhile, Hara et al. [34] also reported that Grade 2 + HT was associated with bone marrow V_5_ ≥ 98% in cervical cancer. Considering that previous studies have concluded the effect of low-dose radiation parameters on total HT, and that the results we did may have been influenced by the small sample size of patients, we preferred limmiting VB V_5_ < 88.75% as a proper dosimetric recommendation to reduce the risk of Grade 3 + HT in patients with GC. The predictive value of V_5_ was emphasized in our study, which may due to: (a) patients in our study were from a prospective clinical trial, all received 45 Gy of radiotherapy and the same chemotherapy regimen and cycles; (b) the principle of target volume delineation was highly consistent and possessed a narrow fluctuation range and a relatively small heterogeneity of VB dosimetric parameters. In daily practice of GC radiotherapy, however, the predictive value of other dosimetric parameters, such as V_10_, V_20_, V_30_, may also be considered, based on the studies referred above.

**Table 5:** Vertebral body dosimetric parameters for some common thoracic and abdominal tumors

Study (Ref.), Year	Tumor type, N	Therapeutic regimen	VB dosimetric thresholds
Wang et al. (31), 2016	Gastric cancer, n = 25	Adjuvant CRT (45 Gy/25f, chemotherapy based on capecitabine)	V_5_ ≤ 90% for Grade 3 + leukopenia; V_20_ ≤ 78% for Grade 3 + neutropenia; V_30_ ≤ 60% for Grade 3 + thrombocytopenia
Zhang et al. (14), 2019	Esophageal cancer, n = 53	Preoperative CRT (41.47 Gy, carboplatin-paclitaxel)	V_10_ ≤ 49.1%, V_20_ ≤ 46.5%, D_mean_ < 17.2 Gy for Grade 3 + leukopenia
Fabian et al. (29), 2019	Esophageal cancer, n = 137	Neoadjuvant or definitive CRT (50.4–59.4 Gy, chemotherapy based on cisplatin/5-fluorouracil)	V_30_ < 14% for Grade 3 + total HTs
Lee et al. (30), 2016	Esophageal cancer, n = 41	Neoadjuvant CRT (41.4–48 Gy, chemotherapy based on cisplatin/ 5-fluorouracil)	V_20_ < 70%, V_10_ < 77%, D_mean_ < 25.9 Gy for Grade 3 + leukopenia
Barney et al. (28), 2017	Lung cancer, n = 201	Definitive CRT (60–70 Gy, chemotherapy based on cisplatin)	V_5_ ≤ 65%, V_10_ ≤ 60%, V_20_ ≤ 50%, D_mean_ ≤ 23.5 Gy for Grade 3 + total HTs
Shaikh et al. (15), 2015	Pancreatic cancer, n = 49	Definitive CRT (chemotherapy based on gemcitabine)	V_5_ < 57.6% for Grade 2 + leukopenia; D_max_ < 48.02 Gy for Grade 2 + neutropenia

CRT, chemoradiotherapy

We delineated VB based on the CT simulation in this study, and designed the delineation protocol which was proved convenient and practical. Mahantshetty et al. [35] proposed to contour the inner cavity of the bone as it could be a better surrogate of the active bone marrow. Some studies used positron emission tomography-computed tomography (PET-CT) or magnetic resonance imaging (MRI) to identify and delineate active bone marrow [31, 36], which caused difficulty to some extent in radiation planning. In the clinical practice, the delineation method based on CT scanning is more commonly used and economical for GC radiotherapy, and the VB dosimetric parameters that we concluded are more clinically instructive than those based on PET-CT or MRI.

To our knowledge, this is the first study to predict acute HT using VB dosimetric parameters in patients with GC receiving nCRT, which excluded the impact of surgery. Studies on lung and esophageal cancers have considered the effect of radiation doses to the ribs, clavicle, sternum, and scapula on bone marrow suppression outside the VB [28, 29], but the effect of these bones on HT was not considered in our patients. Given the lower location of the GC treatment fields, it’s not likely that these bones receiving meaningful low-dose radiation exposure. Meanwhile, we were not able to correlate bone dose-volume metrics with the long-term changes in blood cell counts that may affect the patients’ quality of life, which required a longer follow-up.

## Conclusions

In conclusion, nCRT could increase the risk of Grade 3 + HT compared with nCT. Dose constraints of V_5_ < 88.75% should be considered to reduce the incidence of Grade 3 + HT in patients with GC treated with IMRT, especially when oxaliplatin and capecitabine are used as chemotherapeutic agents.

## Data Availability

All data generated or analyzed during this study are included in this published article.

## Abbreviations

HT: hematological toxicity
GC: gastric cancer
nCRT: neoadjuvant chemoradiotherapy
nCT: neoadjuvant chemotherapy
VB: vertebral body
CTCAE: Common Terminology Criteria for Adverse Events
ROC: receiver operating characteristic
EGJ: esophagogastric junction
CTV: clinical target volume
OAR: organ at risk
EUS: endoscopic ultrasound
CT: computed tomography
DFS: disease-free survival
OS: overall survival
XELOX: capecitabine + oxaliplatin
3D-CRT: three-dimensional conformal radiation therapy
IMRT: intensity-modulated radiation therapy
CT-sim: simulation computed tomography
EORTC-ROC: European Organization for Research and Treatment of Cancer
NCCN: National Comprehensive Cancer Network
GTV: gross tumor volume
PTV: planning target volume
IVD: intervertebral discs
DVH: dose-volume histogram
VBV: vertebral body volume
D_max_: maximum dose
D_mean_: mean dose
D_min_: minimum dose
V_x_: the volume percentage of dose receiving ≥ x Gy
WBC: white blood cell
ANC: absolute neutrophil count
HGB: hemoglobin
PLT: platelet
SD: standard deviation
UVA: univariate logistic regression analysis
MVA: multivariate logistic regression analysis
OR: odds ratio
CI: confidence interval
AUC: area under the curve
PET-CT: positron emission tomography-computed tomography
MRI: magnetic resonance imaging
